# *In vitro* cultured human endometrial cells release extracellular vesicles that can be uptaken by spermatozoa

**DOI:** 10.1038/s41598-020-65517-9

**Published:** 2020-06-01

**Authors:** Valentina Murdica, Elisa Giacomini, Sofia Makieva, Natasa Zarovni, Massimo Candiani, Andrea Salonia, Riccardo Vago, Paola Viganò

**Affiliations:** 1 Urological Research Institute, IRCCS Ospedale San Raffaele, Milan, 20132 Italy; 2 Reproductive Sciences Laboratory, Obstetrics and Gynecology Unit, IRCCS San Raffaele Scientific Institute, Milano, 20132 Italy; 3 Exosomics Siena S.p.A, Siena, 53100 Italy; 4 Università Vita-Salute San Raffaele, Milan, 20132 Italy; 5 Obstetrics and Gynecology Unit, IRCCS San Raffaele Scientific Institute, Milano, 20132 Italy

**Keywords:** Cell biology, Cell biology, Medical research, Medical research

## Abstract

Extracellular vesicles (EVs) derived from different parts of the male reproductive tract can be internalized by human spermatozoa affecting their maturation and regulating their functions. Here we demonstrate that EVs derived from the female tract can be uptaken by sperm and affect their competence. Primary endometrial cells release EVs with a diameter between 50 and 350 nm and bear the standard vesicle and exosome marker proteins CD63, CD9, TSG101 and ALIX. The uptake of dye-labelled endometrial cell-derived EVs by spermatozoa, quantified as fluorescence intensity, was significantly higher when EVs were derived from cells in the proliferative phase. Vital, motile fluorescent sperm could be appreciated after a 48-hour co-incubation with endometrial cells previously labelled with the Vybrant™ DiO dye. EV internalization by sperm was blocked at 4 °C and by incubation with filipin, suggesting an energy-dependent process probably attributable to the lipid-raft domain mediated-endocytosis. Sperm ability to undergo capacitation and acrosome reaction was stimulated by endometrial cell-derived EVs as manifested by the increased protein tyrosine phosphorylation and evident reactivity when stimulated with a calcium ionophore. Based on these findings, EVs exchange may be suggested as an emerging way through which female reproductive tract cells can interact with the passing spermatozoa.

## Introduction

Ejaculated sperm must transit through the female reproductive tract in order to fertilize an egg. The removal of seminal plasma components and the exposure to secretions from the female environment, although for a limited period of time, seem to be critical for inducing sperm capacitation.

The capacitation process is associated with an increase of the membrane structure and fluidity due to a removal of cholesterol from the sperm plasma membrane and to an increase of sperm intracellular calcium and bicarbonate ion permeability^1,2^. These modifications induce the tyrosine phosphorylation of a subset of sperm proteins, which is mediated by the cyclic AMP (cAMP)-dependent protein kinase A^3,4^. Supporting a role of the uterine cells in sperm capacitation, human spermatozoa co-incubated with endometrial cell-conditioned medium showed an increase in protein tyrosine phosphorylation^5^. Although the mechanism undertaking this communication is largely unknown, recent evidence would suggest that extracellular vesicles (EVs) may play a role in this context.

Extracellular vesicles are membranous blebs or vesicles originating from the endosomal system released from most cells as a result of a variety of biological processes. They carry a multitude of molecules, such as receptors, proteins, lipids and nucleic acids including mRNA and miRNA, and thus transfer information to cells in the close proximity of, or at a distance from, parent cells. The interaction between EVs and recipient cells could involve several events including binding of ligands from the EVs to receptors on the recipient cell, fusion of the EV membrane with the recipient cell membrane or the vesicle could be uptaken by the recipient cell by endocytosis.

Franchi and coworkers have recently shown that EVs secreted by endometrial epithelial cells can be uptaken by human spermatozoa^6^. Indeed, sperm cells incubated with fluorescent-labelled EVs derived from endometrial cells acquired the fluorescent staining indicating that EVs might be transferred to sperm. A short co-incubation of spermatozoa with endometrial cell-derived EVs could increase sperm protein tyrosine phosphorylation and the percentage of sperm undergoing acrosome reaction. Even though the study is interesting, the source of EVs was represented by an endometrial adenocarcinoma cell line, which certainly does not mirror the functionality and cyclicity of a normal endometrium^6^. Moreover, the mechanisms for EV uptake by sperm have not been extensively explored. On these bases, the aim of the present study was to gain a better insight into the potential involvement of EVs in mediating the communication between the endometrium and the spermatozoa. Here we show that EVs released by primary endometrial cells can be internalized by spermatozoa with a significant effect on their competence.

## Results

### Isolation and characterization of EVs from human primary endometrial cells

**Figure 1:**
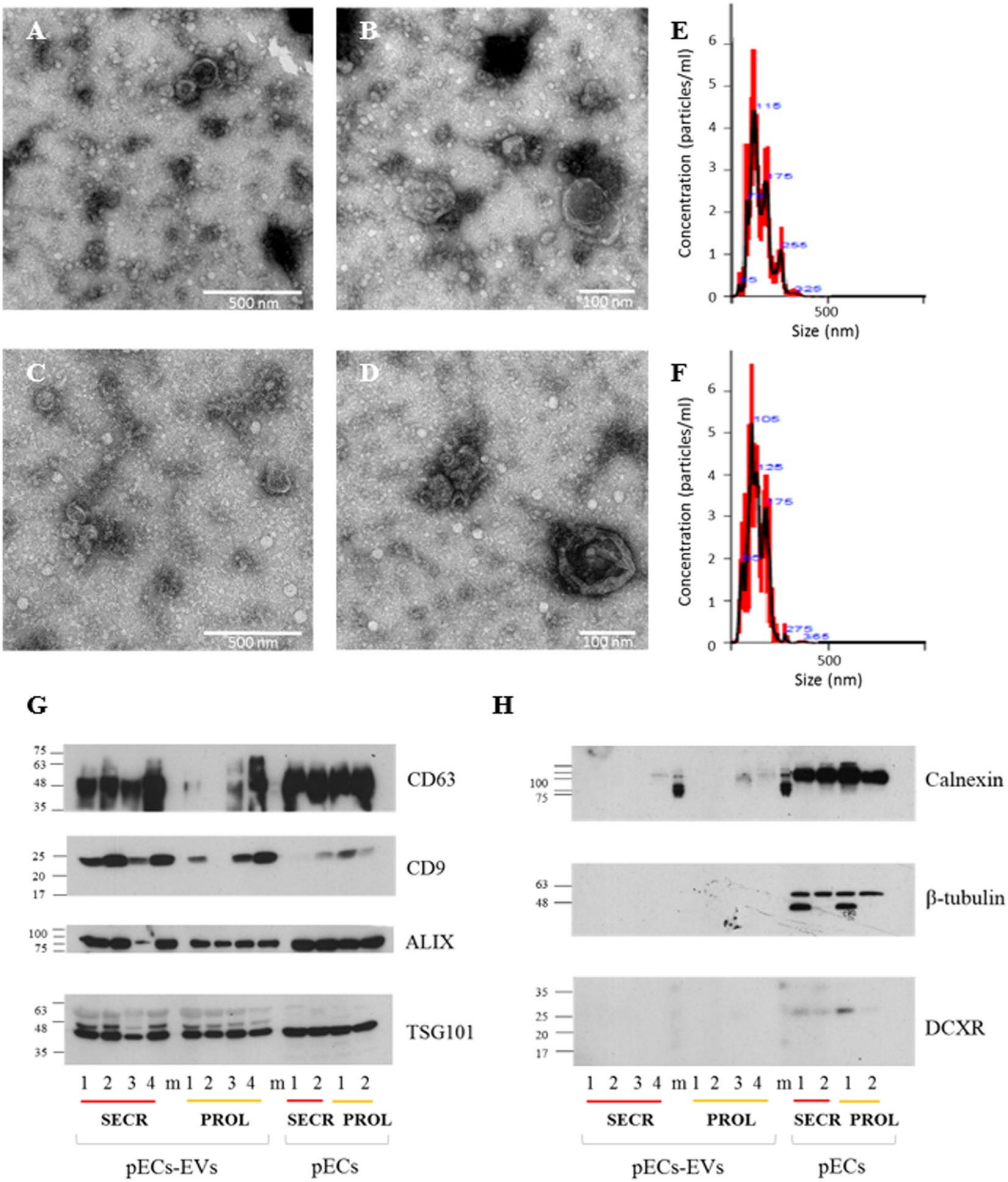
Characterization of pECs-EVs from different women in secretory and proliferative phase of cycle. Representative TEM images of pECs-EVs from secretory (**A,B**) and proliferative phase (**C,D**). Scale bars: 50 nm (**A,C**); 100 nm (B,D). Representative nanoparticle tracking analysis plots of pECs-EVs from secretory (**E**) and proliferative phase (**F**). Western blot showing the presence of different canonical EV markers (CD63, CD9, Alix and TSG101) in secretory and proliferative phase pECs-EVs (n = 4) (**G**). Negative EV markers used were Calnexin (ER), β-tubulin and the cytosolic form of DCXR (**H**). The same markers were evaluated in pECs protein extract as controls for EV enrichment of these markers.

Conditioned media from human primary endometrial cells (pECs) were centrifuged and filtered, then pECs-derived EVs (pECs-EVs) were isolated by ultracentrifugation according to a standard procedure. The size and concentration of pECs-EVs were determined by Transmission Electron Microscopy (TEM) (*n* = 3) and Nanoparticle Tracking Analysis (NTA) (*n* = 3). TEM analysis indicated the presence of double membrane vesicles often with a thin and electron dense membrane. Primary ECs-EVs derived from both secretory (Fig. 1A,B) and proliferative phase (Fig. 1C,D) displayed similar shape, size and electron-density (Fig. 1A–D) and were between 50 and 350 nm in size. In detail, vesicles from both phases peaked between 75 and 150 nm, with the majority being>100 nm. This finding is in line with results from NTA showing similar size distribution profiles for the secretory (Fig. 1E) and proliferative (Fig. 1F) phase EV populations, having a diameter of 143.4 ± 2.7 nm and 136.9 ± 6.9 nm (mean±SEM) respectively (Fig. 1). The average modal size (defined as the most frequently occurring EV size) of pECs-EVs from secretory and proliferative phases were 103.0 ± 14.6 nm and 119.0 ± 14.6 nm (mode±SEM) respectively.

To further confirm that the structures observed in the ultracentrifugation pellet were indeed EVs according to the guidelines of the International Society for Extracellular Vesicles^7^, characterization was also assessed by western blotting analysis. Proteins were isolated, separated and probed with antibodies against standard EV markers (Fig. 1G) and against endoplasmic reticulum-resident protein calnexin, cytosolic proteins tubulin and DCXR as negative controls (Fig. 1H). As expected, vesicles were positive for tetraspanins (CD63 and CD9) and soluble proteins (ALIX and TGS101) (Fig. 1G) but not for the negative controls (Fig. 1H). The immunostaining of total pECs protein extracts with the same antibody was used as control.

### pECs-EVs can be uptaken by sperm and stimulate capacitation

**Figure 2:**
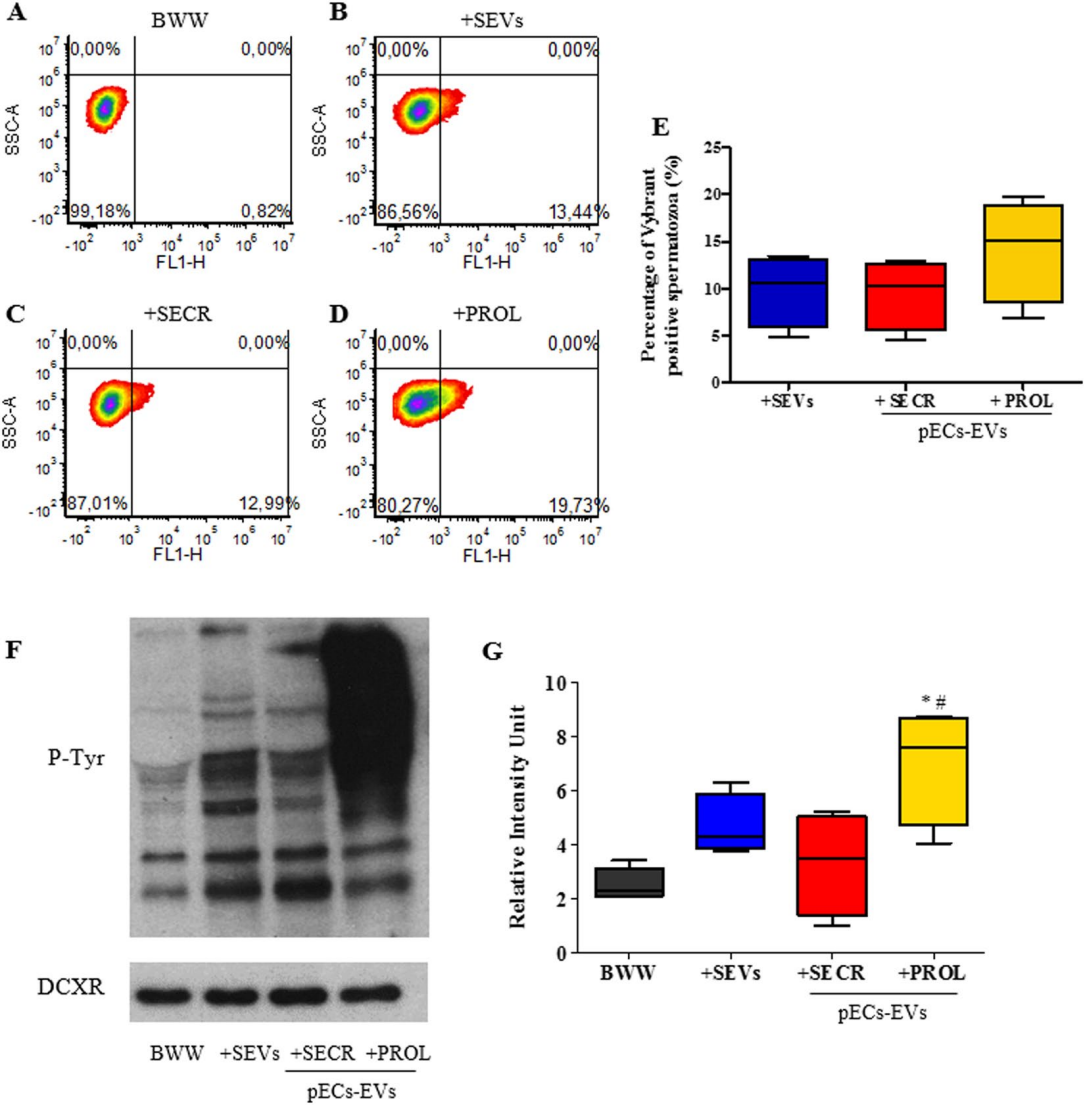
pECs-EVs can be uptaken by spermatozoa and mediate protein tyrosine phosphorylation. *Upper panel*: Spermatozoa were incubated (**B**) with Vybrant DiO-labelled SEVs derived from normozoospermic men (*n* = 4) or with labelled-pECs-EVs from secretory (*n* = 4) (**C**) and proliferative (*n* = 4) (2D) phases for 4 hours. The “pelleted” dye labelled-PBS re-suspended in Biggers Whitten Whittingham (BWW) “non-capacitating” medium was used as negative control (**A**). Sperm incubation was performed in BWW “non-capacitating” medium and EV uptake was assessed by means of flow cytometry analysis. Box and whisker plot shows minimum, 25th percentile, 50th percentile (median), 75th percentile, and maximum values of the percentage of Vybrant DiO positive-spermatozoa. Data represent four independent experiments performed with the use of spermatozoa from different normozoospermic men (**E**). *Lower panel*: Spermatozoa from normozoospermic donors were incubated in BWW “non-capacitating” medium and BWW “non-capacitating” medium supplemented with SEVs from normozoospermic men (*n* = 4) or with pECs-EVs from secretory (*n* = 4) and proliferative phases (*n* = 4). The level of protein tyrosine phosphorylation (pTyr) was analyzed by means of western blot with the use of anti-phosphotyrosine antibody (**F**). The cytosolic form of sperm DCXR protein was used as a loading control. Box and whisker plot shows minimum, 25th percentile, 50th percentile (median), 75th percentile, and maximum values of the quantified bands (**G**). Two-way ANOVA followed by Bonferroni’s post-test was used to determine significance: *p < 0.01 for sperm treated with pECs-EVs from proliferative phase versus control (BWW “non-capacitating” medium) and ^#^p < 0.05 for sperm treated with pECs-EVs from proliferative versus those from secretory phase.

In order to assess whether pECs-EVs can be captured by healthy spermatozoa, purified EVs were labelled with the fluorescein-based, lipophilic Vybrant™ DiO dye and subsequently were incubated with sperm from normozoospermic men (*n* = 4) for 4 hours and analyzed by flow cytometry (Fig. 2 upper panel). In line with our previous data^8^, spermatozoa could capture semen-derived EVs (SEVs) with an uptake efficiency represented by the percentage of green fluorescence positive cells from 4.9% to 13.4% (mean ± SEM: 9.9 ± 1.9%) (Fig. 2B,E). Similar results could be observed after treatment of sperm with secretory phase pECs-EVs as the percentage of positive cells ranged from 4.5% to 13% (mean ± SEM: 9.5 ± 1.9%)(Fig. 2C,E). The treatment of sperm with proliferative phase pECs-EVs resulted in a not statistically significant increase in the uptake efficiency, ranging from 6.9% to 19.7% (mean ± SEM: 14.2 ± 2.7%), supporting some heterogeneity among patients (Fig. 2D,E).

We then tested the possible effect of pECs-EVs on sperm capacitation by assessing sperm protein tyrosine phosphorylation (Fig. 2 lower panel). Sperm cells were treated with basal medium (BWW “non-capacitating medium”^8^) supplemented with EVs and the sperm protein phosphotyrosine level was examined after 4 hours (Fig. 2F). Our group has already demonstrated that the treatment of sperm with SEVs from normozoospermic men can stimulate sperm capacitation with a maximum effect at 4 hours^8^ (Fig. 2F,G). While treatment with secretory phase pECs-EVs showed a similar result, treatment with EVs from the proliferative phase stimulated a significantly higher response (Fig. 2F,G).

**Figure 3:**
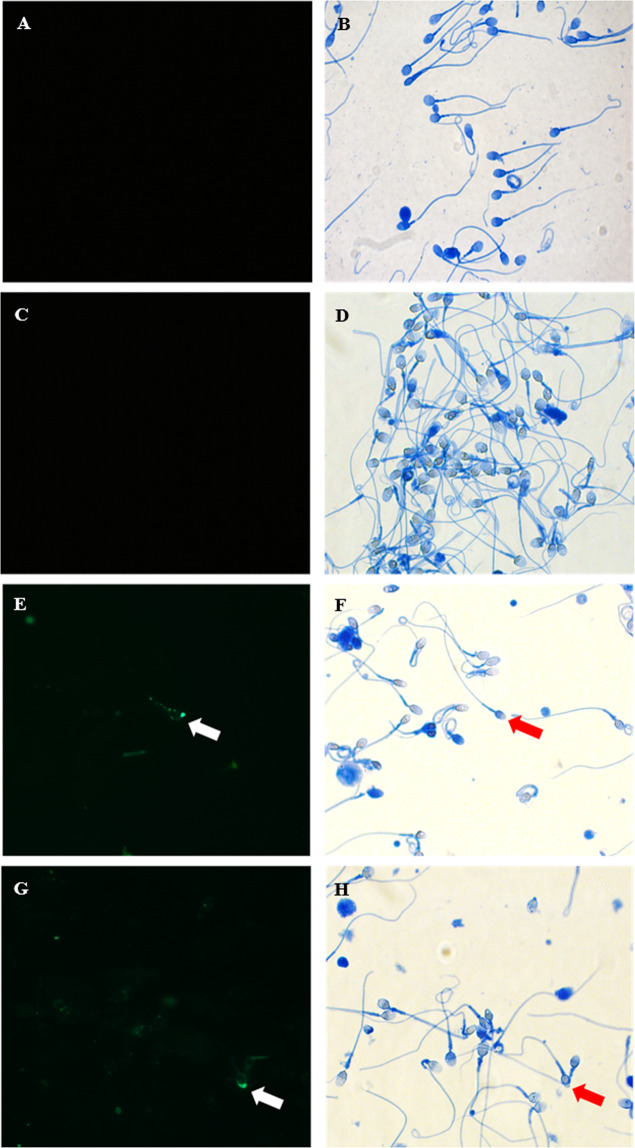
pECs-EVs are uptaken by spermatozoa and favor acrosome reaction. The presence of Vybrant™ positive-acrosome-reacted sperm was investigated by fluorescence microscopy. Sperm was stimulated with calcium ionophore and stained with Coomassie Brilliant Blue after 4 hours of incubation with Vybrant DiO-labelled pECs-EVs in BWW “non capacitating” medium. (**E–H**). Sperm incubated with “pelleted” dye labelled-PBS re-suspended in BWW “non-capacitating” medium (**A,B**) or in BWW “capacitating” medium (**C,D**), were used as negative and positive controls, respectively. Left panels shows fields evaluated at the fluorescent channel (**A–G**) while the corresponding phase-contrast images are shown in the right panels (**B–H**). White arrows indicate Vybrant-positive sperm cells, red arrows indicate the same acrosome reacted/green positive spermatozoa. The acrosome region is stained blue in the acrosome-intact sperm and unstained in the acrosome-reacted sperm. Original magnification 630x.

We also investigated the ability of pECs-EVs to induce the acrosome reaction. For this purpose, incubation of spermatozoa in BWW “non-capacitating medium” supplemented with pECs-EVs was performed for 4 hours. Sperm cells were then stimulated with a calcium ionophore and stained with Coomassie brilliant blue (Fig. 3). The acrosome region was stained blue in acrosome-intact sperm but become unstained in reacted cells, as clearly displayed in the BWW “capacitating medium” condition (Fig. 3C–D). The percentage of sperm that underwent an acrosome reaction after pECs-EVs administration (Fig. 3E–H) was 39.20 ± 3.54%. As negative control, EV untreated cells in BWW “non-capacitating medium” were considered (Fig. 3A,B). Our group has already demonstrated that the treatment of sperm with SEVs from normozoospermic patients can stimulate around 50% of sperm acrosome reaction with a maximum effect at 4 hours^8^.

**Figure 4:**
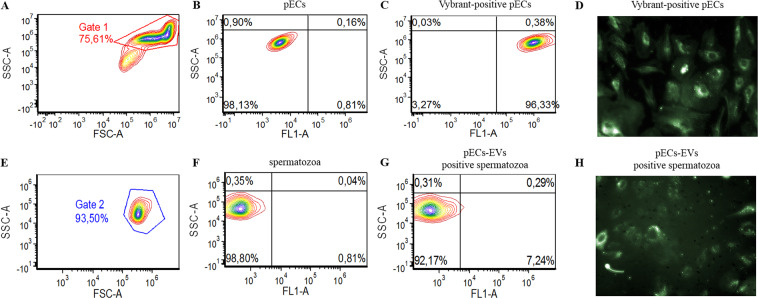
pECs-EVs can be uptaken by spermatozoa in a co-culture system. pECs-EVs were labelled for 2 hours with Vybrant DiO dye, analyzed by flow cytometry (**C**) and live imaging (**D**) and allowed to release EVs for 48 hours. Physical parameters (**A**) and fluorescence (**B**) of unlabeled pECs were analyzed for additional 48 hours (see Materials and Methods section and Supplementary Figure 2). Uptake confirmation and efficiency were evaluated by flow cytometry (**G**) and live imaging snapshot (**H**). Physical parameters (**E**) and fluorescence (**F**) of spermatozoa before incubation with pECs are shown as controls. Sperm cells recovered from the co-culture demonstrated a diffused fluorescence both by flow cytometry (**G**) and live imaging snapshot (**H**).

In order to confirm the uptake of pECs-EVs by sperm, we modelled *in vitro* the microenvironment whereby the spermatozoa encounter endometrial cells in the female reproductive tract by co-culturing human spermatozoa with pECs. Initially, the pECs were labelled with the fluorescent dye Vybrant™ DiO and cultured for 48 hours. Labelled pECs were then co-cultured with spermatozoa for additionally 48 hours (Supplementary Fig. 1). Physical parameters of endometrial cells are shown in Fig. 4A and physical parameters of sperm are shown in Fig. 4E. Non-labelled endometrial cells are shown in Fig. 4B while labelled endometrial cells exhibited a spotted fluorescent pattern by live imaging (Fig. 4D) and a positivity of 96.3% by flow-cytometry analysis (Fig. 4C). We then assessed the pECs-EV uptake efficiency by sperm (Fig. 4G,H, Supplementary Figure 2 and Supplementary movies 1–2). In the co-culture setting, some vital, motile sperm cells demonstrated a diffused fluorescence suggesting an uptake mechanism for EVs released from pECs (Fig. 4H, Supplementary Figure 2 and Supplementary movies 1–2). We therefore evaluated the percentage of green fluorescence positive sperm recovered from the co-culture by flow cytometry, which was 6.97 ± 0.30%.

### Cellular internalization of EVs occurs through lipid raft-mediated endocytosis

**Figure 5:**
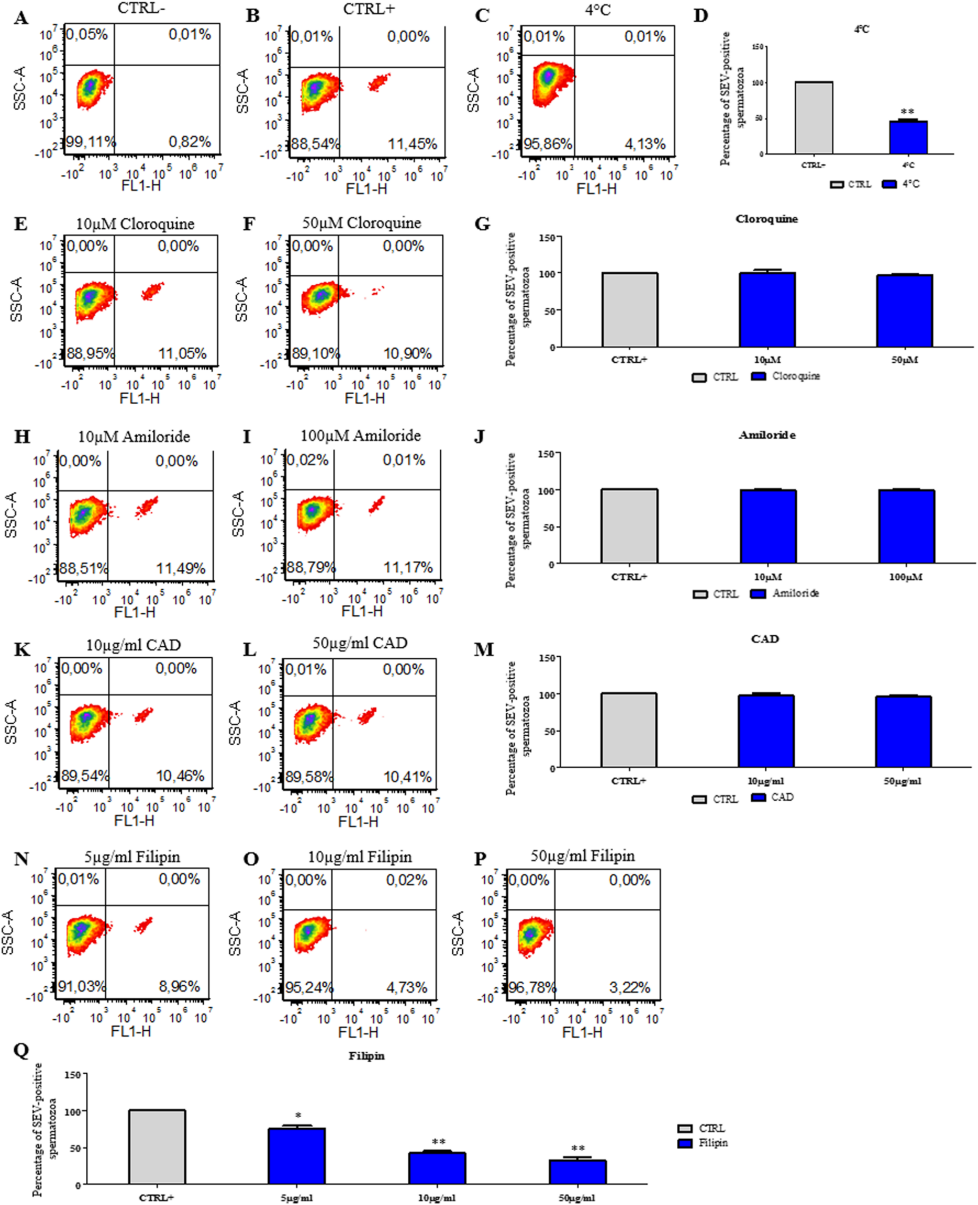
SEVs are internalized by sperm through lipid-raft mediated endocytosis. Sperm cells were incubated in presence (**B–Q**) or absence (**A**) of SEVs. The negative control (**A**) was evaluated by administering to the untreated cells the “pelleted” dye labelled-PBS, while the positive control (**B**) was conducted by adding labelled SEVs to sperm cells not treated with the different drugs. The experiment was performed at 37 °C, with the exception of the 4 °C treatment (**C**), as indicated. SEV uptake by spermatozoa cells in presence or not (CTRL+) of increasing concentrations of cloroquine (**E–G**), amiloride (**H–J**), CAD (**K–M**) or filipin (**N–Q**) was quantified by flow cytometry. Three experiments for each conditions were performed. Values represent mean ± SEM; *p < 0.05, **p < 0.01 versus CTRL+.

**Figure 6:**
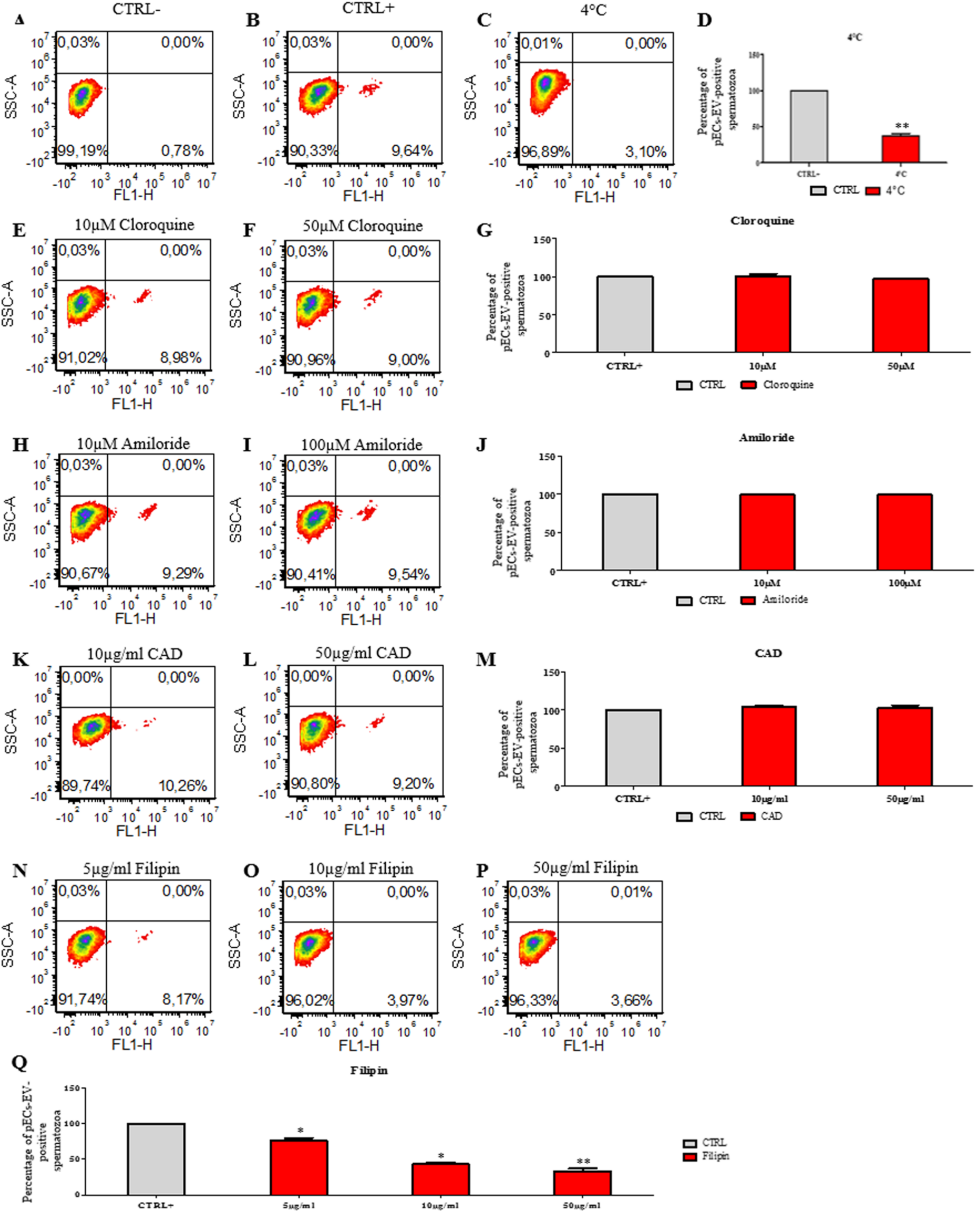
pECs-EVs are internalized by sperm through lipid-raft mediated endocytosis. Sperm cells were incubated in presence (**B–Q**) or absence (**A**) of pECs-EVs. The negative control (**A**) was evaluated by administering to the untreated cells the “pelleted” dye labelled-PBS, while the positive control (**B**) was conducted by adding labelled pECs-EVs to sperm cells not treated with the different drugs. The experiment was performed at 37 °C, with the exception of the 4 °C treatment (**C**), as indicated. pECs-EV uptake by spermatozoa cells in presence or not (CTRL+) of increasing concentrations of cloroquine (**E–G**), amiloride (**H–J**), CAD (**K–M**) or filipin (**N–Q**) was quantified by flow cytometry. Three experiments for each conditions were performed. Values represent mean ± SEM; *p < 0.05, **p < 0.01 versus CTRL+.

The mechanisms accounting for the internalization of EVs in sperm has been also investigated. First of all, a decrease in temperature conditions of the sperm treatment with EVs from 37° to 4 °C caused a reduction of 53.8 ± 2.3% in the number of labeled cells after SEV administration (Fig. 5C,D) as well as a decrease of 62.3 ± 3% in the uptake for the labeled pECs-EVs (Fig. 6C,D). These results indicate that EV uptake by spermatozoa is an energy-dependent process. To identify the mechanisms involved in EV internalization, we tested the efficacy of drugs such as cloroquine, which inhibits autophagy and macropinocytosis in blocking the phenomenon^9,10^. As reported in Figs. 5E,G and 6E,G, cloroquine did not exert any effect on SEV and pECs-EV internalization by spermatozoa. Likewise, amiloride, which is known to block macropinocytosis^11^, either at 10 and 100 μM did not exert any effect on EV uptake in spermatozoa, suggesting that SEVs (Fig. 5H,J) as well pECs-EVs (Fig. 6H,J) were not internalized via macropinocytosis. Treatment with 10 μg/ml and 50 μg/ml of chlorpromazine (CAD), which blocks clathrin-mediated endocytosis^12^, did not show an uptake reduction after both SEV (Fig. 5K,M) and pECs-EV (Fig. 6K,M) administration. Finally, the effect of different concentrations of filipin, a drug inhibiting lipid raft-dependent endocytosis^13,14^, has been tested. We used 5, 10 and 50 μg/ml of filipin, displaying a SEV uptake reduction of 24 ± 3.2%, 57.3 ± 2.4 and 63.5 ± 3.8 respectively (Fig. 5N,K). A similar effect was established for pECs-EV internalization, where a significant fluorescent reduction of 17.1 ± 3.9%, 50.8 ± 6.5 and 58.7 ± 2.2 was observed respectively (Fig. 6N,K).

## Discussion

In animals with internal fertilization, reproduction after mating implies that sperm migrates to the site of fertilization in the female and encounter the mature oocyte. Since the ejaculate generally contains millions of sperm, a strict selection process is put in place in some species in order to ensure that only highly selected sperm finally arrives to the fertilization site. This process could imply the interactions of spermatozoa during the transit in the uterus and the influence of the uterine milieu. In boars, for instance, the phenomenon has been comprehensively studied as the uterus is thought to store spermatozoa before they are allowed to proceed to the oviducts. Moreover, sperm communicate with the surface of endometrium or with endometrial leukocytes to produce pro- and anti-inflammatory cytokines. As a consequence, neutrophils migrate into the uterine lumen and the leukocyte pattern changes in the endometrium. Since lectins on the epithelial cells are known to interact with glycan molecules on the sperm surface, lectins represent the principal candidates to mediate sperm-uterine interaction^15^.

In humans, this phenomenon has received much less attention mostly due to the variable and often small number of sperm recovered from the uterus in old studies^16^. On the other hand, some evidence supported the presence of intrauterine effectors able to stimulate spermatozoa to acquire their functional competence. In 1994, we were able to demonstrate that spermatozoa incubated in the presence of endometrial cell layers underwent acrosome reactions in a significantly higher percentage compared to those not co-cultured^17^. The number of spermatozoa able to penetrate hamster oocytes was also significantly improved after the endometrium-sperm co-culture^17^. Co-incubation of human spermatozoa with conditioned medium by endometrial cells has been shown to positively affect sperm capacitation as evaluated by tyrosine phosphorylation of sperm protein^5^. The cytokine interleukin-6 secreted by the endometrial cells has been shown to represent one of the uterine factors critical to these events^5^.

In the present study, we confirm previous findings^6^ indicating that primary endometrial cells release EVs of heterogeneous morphology that are positive for the EV/specific markers CD63, CD9 and for the exosomal markers TSG101 and ALIX. A previous study performed evaluating the proteomic profile of vesicles from endometrial cells lines has demonstrated that they contain proteins involved in exosome biogenesis, sorting, trafficking and uptake. A consistent amount of proteins were however unique to endometrial vesicles including proteins involved in cytoskeletal organization, cell adhesion and migration. Several enzyme proteins such as ligases, phosphatases, kinases and hydrolases were also present in endometrial vesicles^18^.

Based on our results, these EVs released by endometrial cells could be internalized by spermatozoa as demonstrated by the direct treatment of spermatozoa with fluorescent labelled**-**pECs-EVs and by the co-culture of labelled-pECs with spermatozoa. Indeed, a proportion of green fluorescent sperm could be observed at flow cytometry after treatment with pECs-EVs and motile fluorescent sperm in the medium could be found after the co-culture. Moreover, we have also examined the impact of pECs-EV supplementation on spermatozoa capacitation by assessing sperm protein tyrosine phosphorylation confirming the activation of the phenomenon especially for EVs deriving from proliferative phase samples. Sperm that took up pECs-EVs also demonstrated the ability to undergo acrosome reaction as assessed by staining with Coomassie Brilliant Blue after treatment with a calcium ionophore.

The limited number of sperm able to uptake EVs is not surprising. It is well known from animal studies that of the thousands of sperm in the isthmus, only a few are transported to the ampulla at the time of fertilization. In hamsters, for instance approximately 1 in 10,000 sperm cells reach the site of fertilization. These sperm reaching the ampulla at the time of fertilization are likely to be very fertile^16^. In this context, the fact that only few sperm can uptake EVs may support the idea that the phenomenon has some form of selectivity and is not an unspecific mechanism.

The recent work of Franchi and co-workers describing the incorporation of endometrial cell-derived vesicles by spermatozoa was limited by the fact that an adenocarcinoma endometrial cell line was used as a model, hampering the possibility to assess the impact of the menstrual phase on the observed effects^6^. In line with a plausible more effective role of the EVs in the proliferative phase, older studies have reported that in all women in which sperm was recovered directly from the uterus, the endometrium was always in the late proliferative phase^16^.

To investigate the internalization of EVs in sperm we have used a standard method involving incubation of cells with vesicles labelled with a fluorescent lipophilic dye. To avoid the potential for dye to be transferred by EVs naturally contained in the serum that supplements the culture medium, we used EV-depleted FBS for the lipophilic dye labelling and for the assessment of cellular uptake. In any case, a possible drawback of the lipophilic dye employment is the potential diffusion of these fluorescent molecules from EVs onto the cellular membrane, leading to an internalization pattern that could be caused by physiological recycling instead of EV capturing. However, this seems unlikely, given the numerous studies reporting the activity of molecular inhibitors preventing the uptake of dye-labelled EVs^19^. Another potential limitation of this technique is that we are not presently aware of the number of labelled vesicles incorporated able to allow the visualization of a fluorescent signal. This concept is also supported by the fact that the experiment evaluating sperm protein tyrosine phosphorylation upon incubation with pECs-EVs showed in general much clearer results than the experiments performed with the use of the lipophilic dye.

In this study, we have used spermatozoa deriving from normozoospermic men attending an Infertility Clinic. However, the diagnosis of normozoospermia does not rule out fertility issues. It has to be underlined that the men selected were partners of women with a known infertility factor. The present findings should be confirmed further using sperm from fertile men for the uptake experiments.

Increasing evidence supports the involvement of EVs in intracellular communication and it is emerging that they have peculiar functions in signalling, waste management, coagulation and inflammation. We have recently demonstrated that even freshly ejaculated spermatozoa can still uptake semen EVs but the molecular events underlying this interaction have not been investigated in depth. Several mechanisms for EV internalization have been proposed and are well reviewed in the literature^19–21^, including clathrin mediated endocytosis^12,22^, phagocytosis^23^, macropinocytosis^12,24^ and plasma^25–30^ or endosomal membrane fusion. The roles of lipid rafts^24,30–33^ have also been investigated. In our study, we used a range of inhibitors to block specific mechanisms involved in these phenomena. Chloroquine is a lysosomotropic agent able to accumulate inside the cell and to prevent endosomal acidification^9^, resulting in the inhibition of the activity of lysosomal enzymes that require an acidic pH, and thus inhibiting the fusion between endosomes and lysosomes^34,35^. Moreover, raising the lysosomal pH, cloroquine inhibits autophagy and hinders the fusion of autophagosome with lysosome^10^. We found that cloroquine did not exert any effect on SEV and pECs-EV internalization by spermatozoa. Another mechanism implicated in EV internalization is represented by macropinocytosis, consisting in an actin-mediated endocytic uptake pathway involving an extension of plasma membrane ruffles, which then pinch off into the intracellular compartments. The alkalinizing compounds bafilomycin, cloroquine and amiloride inhibited microglial internalization of EVs^11^, but not SEV and pECs-EV internalization by spermatozoa, confirming results from other studies that did not suggest a role for macropinocytosis in the uptake of EVs^20,23,31^. Various studies have also implicated clathrin-dependent endocytosis in the uptake of EVs^23,35,36^. Chlorpromazine prevents clathrin-dependent endocytosis and has been shown to determine a reduction in EV uptake by ovarian cancer cells^12^, but not in spermatozoa. Finally, to investigate the role of lipid rafts in EV uptake, filipin-mediated inhibition has been employed. This drug disrupts lipid rafts-mediated endocytosis and caveolar structure and function^13,14^. In spermatozoa treated with filipin, the uptake of labelled-SEVs and -pECs-EVs was strongly inhibited. The response was concentration-dependent and reached 70% of inhibition at the highest dose administered.

Another potential internalization mechanism of EV cargos into cells is through a direct fusion of EV membrane with cellular plasma membrane^25^. Despite the fact that the transfer of epididymosomal proteins to spermatozoa have not been clarified yet, it is a matter of fact that epididymosomes contain domains rich in cholesterol and sphingomyelin, which can directly exchange proteins with the analogous lipid rafts on the surface of spermatooa^29^. Epididymosomes have been also demonstrated to be able to directly fuse with the sperm membrane to change both the protein and lipid composition of sperm^30^. On the other hand, we found that EV internalization was inhibited at 4 °C, indicating that the process is active and energy-dependent, as opposed to passive membrane fusion. All together, these results support the idea that spermatozoa internalized EVs by lipid-rafts domain mediated-endocytosis.

In conclusion, our results strongly support the evidence that EVs released in the uterine tract may be connected to the molecular mechanisms involved in sperm selection before fertilization. The relevance of these events in terms of fertilization efficacy and reproductive outcomes remains to be established.

## Materials and Methods

### Subjects

The study was approved by the Institutional ethical committee (Comitato etico IRCCS Ospedale San Raffaele – protocol “Mummy sperm”, approved on 04/07/2016) and subjects involved provided written informed consent. Twenty patients, aged 18–47 years, undergoing semen analysis at a single IVF academic center were enrolled for the specific purpose of the project. In detail, twenty patients were used for motile sperm isolation and eight for isolation of semen-derived EVs. Patients underwent at least two consecutive semen analyses, both showing standard values for normal semen parameters according to the WHO criteria^36^. Exclusion criteria included diagnosis of varicocele, genitourinary inflammation, seminal tract infections and smoking. Men selected were partners of women with a known infertility factor including endometriosis, reduced ovarian reserve and PCOS.

Human endometrial samples were obtained from women undergoing laparoscopy for ovarian benign pathology using a surgical curette. Women with uterine disorders or who had received steroid hormone treatment in the last three months were excluded from the study. All women were younger than 40 years and had regular menstrual cycles. Menstrual cycle phase was confirmed by histological dating. All the experiments were performed in accordance to the principles set out in the World Medical Association Declaration of Helsinki.

### Semen sample collection and processing

Sperm samples were obtained after almost 2 days of sexual abstinence, allowed to liquefy for 30 minutes at 37 °C, and immediately processed according to the WHO 2010 guidelines^37,38^. A discontinuous Percoll gradient (Percoll ™, GE Healthcare Bio-Sciences AB, Sweden) in PBS was used to separate spermatozoa from the seminal plasma, in order to avoid capacitating conditions and incubated in BWW “non-capacitating” medium without human serum albumin and NaHCO_3_, as previously described^8^. Afterwards, the motile sperm population was incubated at 37 °C in 5% CO_2_ in air.

### Primary endometrial cell isolation and culture

Primary endometrial cells (pECs) were obtained from endometrial curettage as previously described^39^.

### Isolation of EVs

EVs were isolated from seminal plasma following the previously reported protocol^8^.

Conditioned media were harvested from pECs and centrifuged at 500 × g at 24 °C for 25 minutes to remove detached cells. Supernatants were collected and filtered through 0.22 µm filters (Merck Millipore) to remove contaminating apoptotic bodies, microvesicles  and cell debris. Clarified media was then centrifuged in a Sorvall WX Ultracentrifuge (ThermoFisher Scientific, WX Ultra 100 #75000100) at 150.000 × g at 4 °C for 90 minutes with a SureSpin 630 swinging bucket rotor (ThermoFisher Scientific) to pellet EVs. The supernatant was carefully removed, and EV-containing pellet were re-suspended in PBS and stored at −80 °C until use.

### Nanoparticle tracking analysis

Size distribution and quantity of EVs were assessed by NTA and performed using a NanoSight LM10-HS microscope (NanoSight Ltd., Amesbury, UK), as previously described^8,39^. The NanoSight system was calibrated as previously described^8^.

### Transmission electron microscopy

EVs derived from pECs cultured media were freshly purified and absorbed on glow discharged carbon coated formvar copper grids. The procedure was then performed as described previously^8^.

### Western blot analysis

Isolated EVs were re-suspended in Laemmli buffer and boiled for 5 minutes at 95 °C. Specifically, 20 µg of EVs were prepared in non-reducing conditions for tetraspanins detection, while 35 µg were used for soluble protein detection. Proteins were separated by SDS-PAGE and transferred onto a nitrocellulose membrane. Nonspecific sites were saturated with 5% (w/v) skimmed milk in Tris-buffered saline: 150 mM NaCl, 20 mM TrisHCl, pH 7.4, and 0.5% Tween 20 (T-TBS). Membranes were incubated overnight at 4 °C with anti-CD9 (1:1000, BD Pharmingen, #555370, San Jose, CA, USA), anti-CD63 (1:2000; BD Pharmingen, #556019, San Jose, CA, USA), anti-Alix (1:500, Santa Cruz, #sc-271975, Santa Cruz, CA, USA), anti-TSG101 (1:500, Novus Bio, #NB200–112, Littleton, CO, USA), anti-DCXR (1:1000, Abcam, #ab110283, Abcam Inc., Cambridge, UK), anti-Calnexin (1:1000, Sigma, #C7617, Sigma-Aldrich, St. Louis, MO, USA) and anti-β-tubulin (1:5000, Santa Cruz, #sc-9104, Santa Cruz, CA, USA). After washing with T-TBS, membranes were incubated with the horseradish peroxidase-conjugated secondary antibodies diluted 1:2000 for 45 min. Immunoreactive bands were revealed by the enhanced chemiluminescence method (ImmobilonHRP substrate, #WBKLS0500, Millipore Corp., Billerica, MA, USA).

### EV administration to spermatozoa

Extracellular vesicles derived from semen from normozoospermic men were administered at a concentration of 50 µg in BWW “non capacitating” medium per 2.5 × 10^6^ spermatozoa at pH = 7.0. In the same way, 50 µg of EVs derived from pECs in secretory or proliferative phase were used to treat spermatozoa. EVs from various semen samples or pECs were used to treat a single sperm sample at the time. All the parameters evaluated in these studies, from the EV uptake to the protein tyrosine phosphorylation, were performed in such experimental conditions.

### EV fluorescence labelling

Extracellular vesicles were labelled with the green Vybrant™ DiO cell-labelling solution (#V22886, Molecular Probes, Eugene, OR, USA), as previously reported^40^, with some modifications. Briefly, 0.5 µl of dye was diluted in 100 µl of PBS and added to 50 µg/100 µl of EV suspension or to 100 µl of PBS only for negative control for 30 min at 37 °C by rotation. The excess of dye was removed by centrifugation at 150.000 × g for 1 hour at 4 °C and the pelleted Vybrant™ DiO-labeled EVs or “pelleted” dye labelled-PBS, re-suspended in BWW “non capacitating” medium, were added to 2.5–3 × 10^6^ spermatozoa at 37 °C. EV uptake was evaluated after 4 hours by flow cytometry analysis. Spermatozoa were washed twice with PBS and analyzed by FACS BD Accuri C6 Plus (BD Bioscience). For each sample, twenty thousand events were acquired. Data were recorded with BD FacsDiva (Becton Dickinson) software and analyzed by using FCS Express 6 (DeNovo) Software.

### Protein tyrosine phosphorylation

Spermatozoa were incubated with BWW “non capacitating” medium or BWW “capacitating” medium supplemented with SEVs from normozoospermic men or pECs-EVs from secretory and proliferative phase for 4 hours. After incubation, spermatozoa were washed with PBS and lysed as previously described^8^. Protein concentration measure and tyrosine phosphorylation analysis was performed as previously reported^8^.

### Induced acrosome reaction

In order to examine the EV-mediated acrosome reaction, spermatozoa were incubated with BWW medium supplemented with labeled ECs-EVs for 4 hours and treated as previously reported (see Materials and Methods section “EVs fluorescence labeling”)^8,41^. Negative control was represented by sperm cells incubated with “pelleted” dye labelled-PBS resuspended in BWW “non-capacitating” medium, while positive control was represented by sperm cells incubated with “pelleted” dye labelled-PBS re-suspended in BWW “capacitating” medium as a technical control of acrosome reacted sperm in the absence of EVs. At the end of the incubation, sperm suspension was stimulated with calcium ionophore A23187 and stained with Coomassie Brilliant Blue following the standard protocol^8,41,42^. The presence of Vybrant™ positive/acrosome-reacted sperm was investigated by fluorescence microscopy. Percentage of whole reacted and non-reacted sperm were assessed by double-blinded counting of 250 spermatozoa.

### Endometrial cells and spermatozoa co-culture: EV uptake assay

Primary ECs were labelled with the Vybrant™ DiO cell-labelling solution (#V22886, Molecular Probes, Eugene, OR, USA) following the manufacturer’s recommendations with some modifications. Briefly, 1.6 × 10^5^pECs plated on 35 mm dishes were washed twice with PBS and fresh RPMI-1640 (without FBS) containing fluorescent dye (5 µl/ml) was added to the pECs for 2 hours at 37 °C. After incubation, cells were thoroughly washed (at least three times) with PBS in order to remove free dye. For controls we used both non-labelled endometrial cells and endometrial cells dye labeled only for 2 minutes. More in detail, for the non-labelled endometrial cells, pECs received only the medium as a basal condition for pECs culture. For the 2 minutes dye labeled endometrial cells, we treated pECs with Vybrant dye for 2 minutes in order to evaluate the unspecific signal. Indeed, this incubation time is insufficient to label pECs and any fluorescence signal obtained using this condition would be due to the unbound dye. Quinn’s Advantage™ Fertilization medium (CooperSurgical Fertility & Genomic Solution, Målov, Denmark), supplemented with 10% FBS (depleted in EVs) and 1% penicillin/streptomycin, was added to labelled-pECs. After 48 h hours, fluorescent pECs were co-incubated with 3 × 10^6^ sperm cells. pECs and sperm co-cultures were maintained at 37 °C for 48 hours. pECs-EV internalization by sperm cells was observed by fluorescence microscopy and a time-lapse movie was generated with the Axio Vision Imaging Software (AxiovisionRel 4.8) on an Axio Imager M2 microscope (Carl Zeiss, Oberkochen, Germany). EV uptake was also investigated at 48 hours by flow cytometry analysis as described above.

### EV uptake inhibition studies

To study the mechanisms of EV uptake, spermatozoa were incubated with several pharmacological/chemical inhibitory molecules before EV supplementation. Cloroquine (10–50 μm) (#AK116457, Ark Pharm, Inc., Arlington Heights, USA), amiloride (10–100 μm) (#A7410, Sigma, Saint Louis, USA), filipin (1–50 μg/ml) (#F4767, Sigma, Saint Louis, USA), or CAD (5–10 μg/ml) (#C8138, Sigma, Saint Louis, USA) were used to treat cells at 37 °C for 30 min prior to EVs addition and were left for the entire duration of the experiments. After the treatment, 20 µg of Vybrant DiO-labeled EVs or “pelleted” dye labelled-PBS were added per million of spermatozoa, and subsequently kept at 37 °C or 4 °C for 4 h. As controls, no treated cells were co-incubated with SEVs or pECs-EVs (positive control) and untreated sperm cells were cultured in absence of SEVs or pECs-EVs (negative control) at the same temperature condition. After washing with PBS, EV uptake was evaluated by flow cytometry analysis as described above.

### Statistical analysis

At least three independent biological replicates were performed for each experiment. Two-way ANOVA test followed by Bonferroni’s post-tests was used to determine significance. All results were expressed as mean ± SEM. Values of p < 0.05 were considered statistically significant.

## Supplementary information

Supplementary information

Supplementary information2

Supplementary information3
