# Supplementary material for: In vitro cultured human endometrial cells release extracellular vesicles that can be uptaken by spermatozoa
Source: Sci Rep. 2020 Jun 1;10:8856. doi: 10.1038/s41598-020-65517-9 (PMC7264351; doi:10.1038/s41598-020-65517-9)
Supplement: Supplementary file 1 — Supplementary information [file 41598_2020_65517_MOESM1_ESM.pdf]

***In vitro* cultured human endometrial cells release extracellular vesicles that can be uptaken by spermatozoa**

Valentina Murdica[1], Elisa Giacomini[2], Sofia Makieva[2], Natasa Zarovni[3], Massimo Candiani[4,5], Andrea Salonia[1,4], Riccardo Vago[1,4]*, and Paola Viganò[2,*]

[1] Urological Research Institute, IRCCS Ospedale San Raffaele, Milan, 20132, Italy

[2] Reproductive Sciences Laboratory, Division of Genetics and Cell Biology, IRCCS San Raffaele Scientific Institute, Milano,20132, Italy.

[3] Exosomics Siena S.p.A, Siena, 53100, Italy

[4] Università Vita-Salute San Raffaele, Milan, 20132, Italy

[5] Obstetrics and Gynecology Unit, IRCCS San Raffaele Scientific Institute, Milano,20132, Italy.

*corresponding: vigano.paola@hsr.it; vago.riccardo@hsr.it

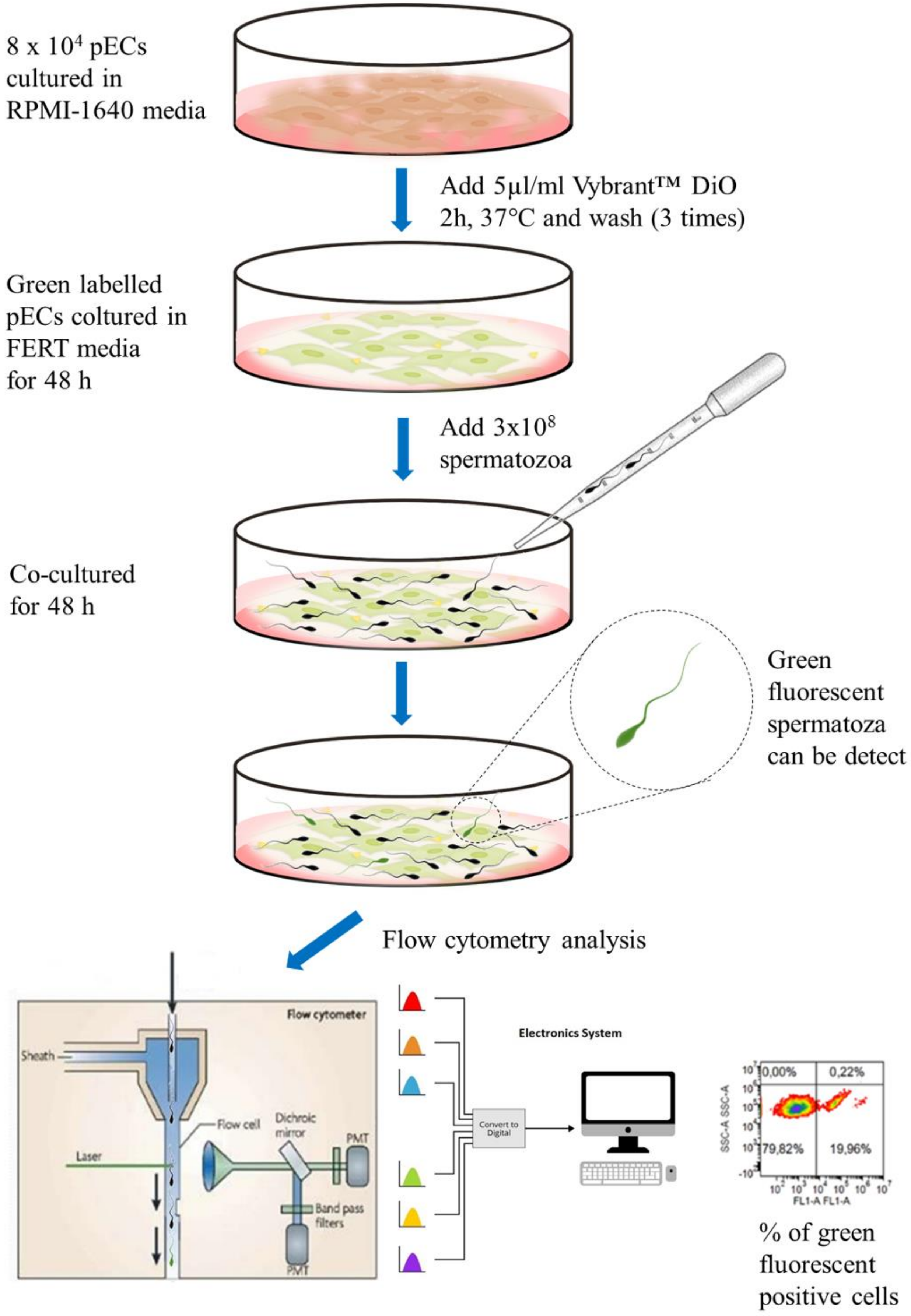

Supplementary Figure 1. Schematic overview of the protocol used to assay pECs-EVs uptake in endometrial cells and spermatozoa co-culture system.

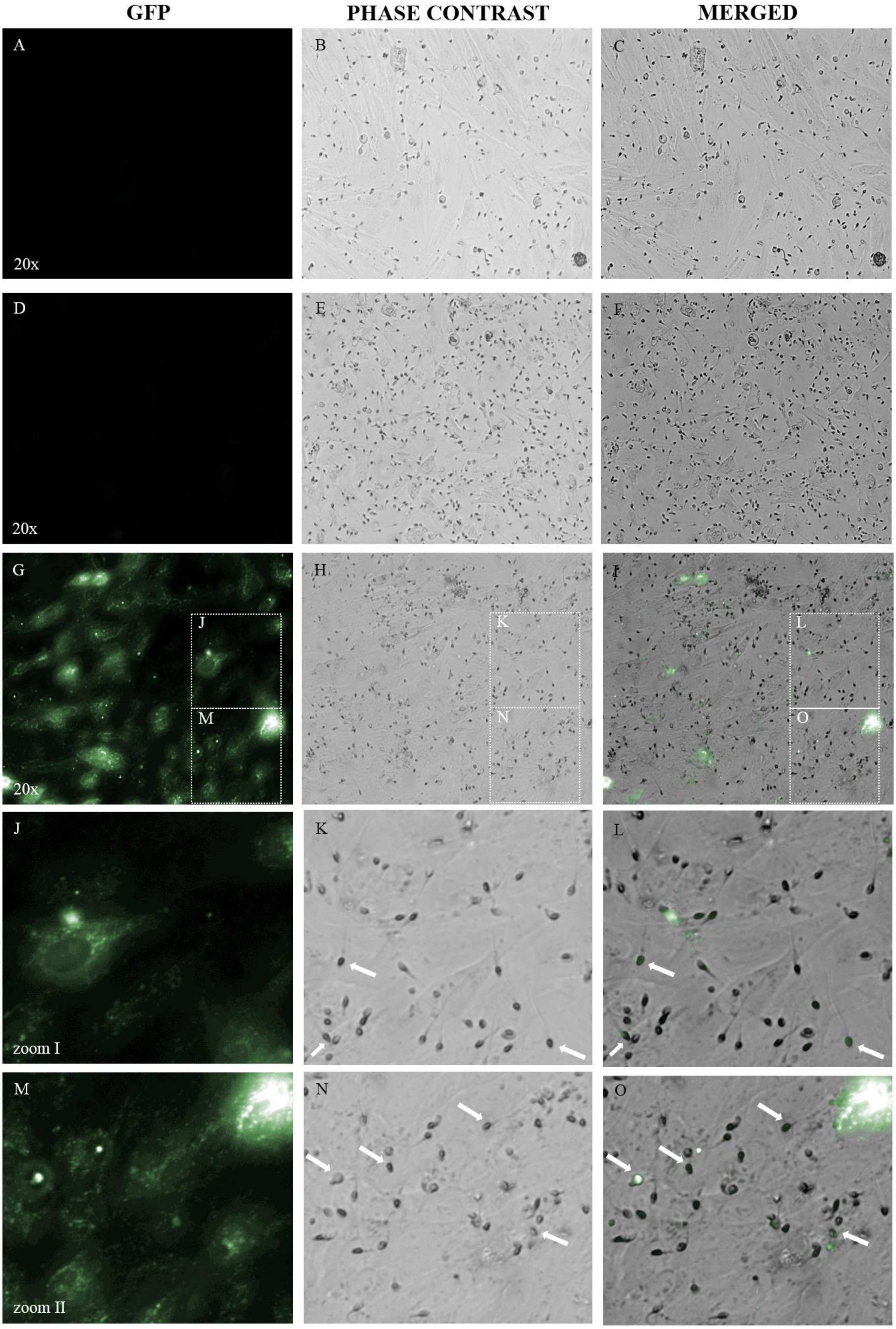

Supplementary Figure 2. pECs-EVs can be taken up by spermatozoa in a co-culture system. pECs were labelled for 2 hours (G-O) with Vybrant DiO dye and allowed to release EVs, as indicated. As negative control, pECs were labeled for 2 minutes (D-F) or unlabelled (A-C). After 48 hours, pECs were co-incubated with 3x10^{6} spermatozoa. EVs uptake was investigated by live imaging after further 48 hours. Figures J-O are the full view of the cells imaged in the inset G-I. White arrows indicate Vybrant-positive sperm cells.
